# Long non-coding RNAs: novel prognostic biomarkers for liver metastases in patients with early stage colorectal cancer

**DOI:** 10.18632/oncotarget.10416

**Published:** 2016-07-06

**Authors:** Hui Kong, Ying Wu, Mengou Zhu, Changwen Zhai, Jing Qian, Xue Gao, Shuyang Wang, Yingyong Hou, Shaohua Lu, Hongguang Zhu

**Affiliations:** ^1^ Department of Pathology, Shanghai Medical College, Fudan University, Shanghai, China; ^2^ Harvard College, Harvard University, Cambridge, MA, USA; ^3^ Department of Pathology, Zhongshan Hospital, Fudan University, Shanghai, China; ^4^ Division of Surgical Pathology, Huashan Hospital, Fudan University, Shanghai, China

**Keywords:** prognostic biomarker, long non-coding RNA, early stage colorectal cancer, liver metastasis, quantitative RT-PCR

## Abstract

Liver metastasis is the primary cause of death for colorectal cancer (CRC) patients. To investigate the prognostic value of long non-coding RNAs (lncRNAs) on colorectal liver metastases, quantitative reverse-transcriptase PCR (quantitative RT-PCR) was performed on 15 lncRNAs in 51 stage IV CRC with liver metastases and 57 stage I/II CRC specimens. The expression levels of four lncRNAs (GAS5, H19, MEG3 and Yiya) were significantly different between liver metastases and primary tumors of stage IV CRC patients. Furthermore, the high expression levels of GAS5 and Yiya were significantly associated with future occurrence of liver metastases in early stage CRC patients. Kaplan-Meier analysis showed that the high expression levels of GAS5 or Yiya were correlated with poor prognosis of early stage CRC patients (p = 0.0206 and 0.0005 for GAS5 and Yiya, respectively). Yiya expression was proved to be an independent prognostic indicator of colorectal liver metastases in a multivariate analysis (relative risk = 10.7; p < 0.0001). Our study revealed that GAS5 and Yiya were promising prognostic biomarkers of liver metastases for early stage CRC patients.

## INTRODUCTION

Colorectal cancer (CRC) is the third most common cancer worldwide, with an estimated 1.4 million cases and 693,900 deaths occurring in 2012 [1]. Metastatic disease is the primary cause of death for CRC patients [2]. According to official statistics, the five-year survival rate for localized CRC patients is up to 90% [3], while it is only 6% for stage IV CRC with distant metastases [4]. Liver is the most common site of metastatic lesions due to the portal drainage. Up to 20% of CRC patients have concurrent hepatic metastases and the five-year cumulative hepatic metastases rate is 14.5% [5, 6].

Clinically, survival time of early stage CRC patients without lymph node or distant metastases varies widely after surgical resections. Most patients do not present hepatic metastases within five years while the others show metastatic deposits in liver soon. This observation emphasizes the clinical and molecular heterogeneity in CRC [4]. Therefore, finding out the factors involved in this heterogeneity will assist clinical intervention and treatment against liver metastases for early stage CRC patients.

Long non-coding RNAs (lncRNAs), typically >200bp, are nature prognostic biomarkers in indicating the intrinsic characteristics of cancer metastasis since they are the effector molecules [7]. A growing body of evidence suggests that lncRNAs are associated with carcinogenesis, tumor metastasis and therapy [8–12]. In addition, clinical application of lncRNAs in cancer prognosis has also made inspiring progress [7, 13]. Nonetheless, little is known about the correlation between lncRNAs and colorectal liver metastases. Besides, whether lncRNAs are involved in the poor prognosis of CRC patients without metastasis has not been investigated yet. To fill up this gap, we evaluated the prognostic values of lncRNAs on liver metastases for patients with early stage CRC.

As lncRNA expression spectrums are often overlapped across different types of cancer, lncRNAs associated with at least two types of cancer are likely to be involved in colorectal liver metastases. Therefore, we collected 15 such lncRNAs (CCAT1, GAS5, H19, HOTAIR, IGF2-AS, lncRNA-LET, MALAT1, MEG3, MIR17HG, p15AS, PANDAR, PVT1, UCA1, XIST and Yiya) from public databases and literature [8, 11, 14–27]. Using quantitative RT-PCR with three non-overlapping short amplicons [28], we measured the expression levels of the 15 collected lncRNAs in stage IV CRC specimens together with liver metastases and identified four candidates for predicting colorectal liver metastases, which were GAS5, H19, MEG3 and Yiya. Furthermore, we validated these candidates by comparing their expression levels between stage I/II CRC patients who presented liver metastases within five years and who did not. Our results revealed that GAS5 and Yiya were promising prognostic biomarkers of liver metastases for patients with early stage CRC.

## RESULTS

### Quantification correlation of short amplicons in 15 lncRNAs

Quantification correlation of three non-overlapping short amplicons for each of the 15 collected lncRNAs and *β-actin* gene was determined in 51 stage IV CRC specimens and corresponding liver metastases (Supplementary Table S1). A strong correlation amongst three amplicons for every lncRNA was achieved in CRC and liver metastases (Spearman rank correlation coefficient >0.70). However, HOTAIR and PANDAR were excluded from this study due to the low expression levels (Cq value >35 in more than 20% samples). Finally, 13 lncRNAs were left for the analysis of association with colorectal liver metastases.

### Association of 13 lncRNAs with colorectal liver metastases

Expression levels of 13 lncRNAs were compared between primary stage IV CRC and matched liver metastases in 51 pairs of samples (Table 1). Paired samples were from the same patient to eliminate individual differences. The expression levels of GAS5, H19 and Yiya were up-regulated in liver metastases compared with primary CRC (fold change = 0.4, 1.0 and 0.6, respectively; all p < 0.0500), while MEG3 was down-regulated (fold change = −0.3; p < 0.0010). Scatter-plots showed the differential expressions of these four lncRNAs between primary CRC and liver metastases (Supplementary Figure S1). The areas under the receiver operating characteristic curve (AUC; represents discrimination accuracy) were 0.68, 0.64, 0.63 and 0.73 for GAS5, H19, Yiya and MEG3, respectively. Thus, these four lncRNAs were further assessed on their prognostic values on live metastases for early stage CRC patients.

**Table 1:** Expression profiles of 13 lncRNAs in stage IV CRC patients compared with matched liver metastases

	Liver metastases / colorectal carcinoma			
	Fold change	P value	AUC	95% CI
CCAT1	0.4	0.1004	0.59	0.49 - 0.69
GAS5	0.4	0.0038	0.68	0.58 - 0.77
H19	1.0	0.0314	0.64	0.54 - 0.73
IGF2-AS	−0.5	0.3582	0.59	0.49 - 0.69
lncRNA-LET	−0.1	0.5322	0.57	0.47 - 0.67
MALAT1	0	0.7225	0.58	0.48 - 0.68
MEG3	−0.3	0.0003	0.73	0.64 - 0.82
MIR17HG	−0.2	0.3072	0.57	0.46 - 0.67
p15AS	0.2	0.7194	0.58	0.48 - 0.68
PVT1	0.2	0.2114	0.54	0.44 - 0.64
UCA1	0.2	0.4989	0.52	0.42 - 0.62
XIST	−0.6	0.3485	0.54	0.44 - 0.64
Yiya	0.6	0.0272	0.63	0.53 - 0.72

CRC, colorectal cancer

AUC, area under the receiver operating characteristic curve

CI, confidence interval

### Association of four lncRNAs with liver metastases in early stage CRC

An independent cohort of 57 patients with stage I/II CRC was divided into two groups according to the presence (n = 21) or absence (n = 36) of liver metastases within five years after surgery (Table 2). Quantitative RT-PCR results of these two groups were shown in Table 3. Higher expression levels of the four lncRNAs were observed in the group presenting liver metastases. However, only the differential expressions of GAS5 and Yiya were statistically significant (fold change = 0.5 and 3.0 for GAS5 and Yiya, respectively; both p < 0.0500, Supplementary Figure S2). The AUC values were 0.65 and 0.70 for GAS5 and Yiya, respectively. The results indicated that the high expression levels of GAS5 and Yiya stimulated liver metastases in early stage CRC patients.

**Table 2:** Characteristics of study subjects

	Variable	Selection phase Identification phase	Validation phase
Patient		51	57
Gender			
	Male	30	30
	Female	21	27
Age (year)			
	Median (range)	61 (35-77)	63 (32-81)
Tissue type			
	Colon	38	29
	Rectum	13	25
	Colorectum	0	3
	Liver metastasis	51	0
TNM stage			
	Tumor stage (T)		
	Tis	0	0
	T1	0	2
	T2	3	18
	T3	5	13
	T4	43	24
	Nodal status (N)		
	N0	15	57
	N1	20	0
	N2	16	0
	Distant metastases (M)		
	M0	0	57
	M1	51	0
Pathological stage			
	0	0	0
	I	0	20
	II	0	37
	III	0	0
	IV	51	0
Histologic grade			
	I	0	1
	II	29	42
	III	22	14
Liver metastases within five years			
	Presence	51	21
	Absence	0	36

**Table 3:** Expression profiles of candidate lncRNAs between stage I/II CRC patients with and without liver metastases

	Presence / absence of liver metastases			
	Fold change	P value	AUC	95% CI
GAS5	0.5	0.0486	0.65	0.51 - 0.77
H19	0.4	0.4178	0.56	0.43 - 0.69
MEG3	0.5	0.1276	0.62	0.48 - 0.74
Yiya	3.0	0.0103	0.70	0.56 - 0.81

CRC, colorectal cancer

AUC, area under the receiver operating characteristic curve

CI, confidence interval

### Prognostic value of four lncRNAs in early stage CRC

**Figure 1:**
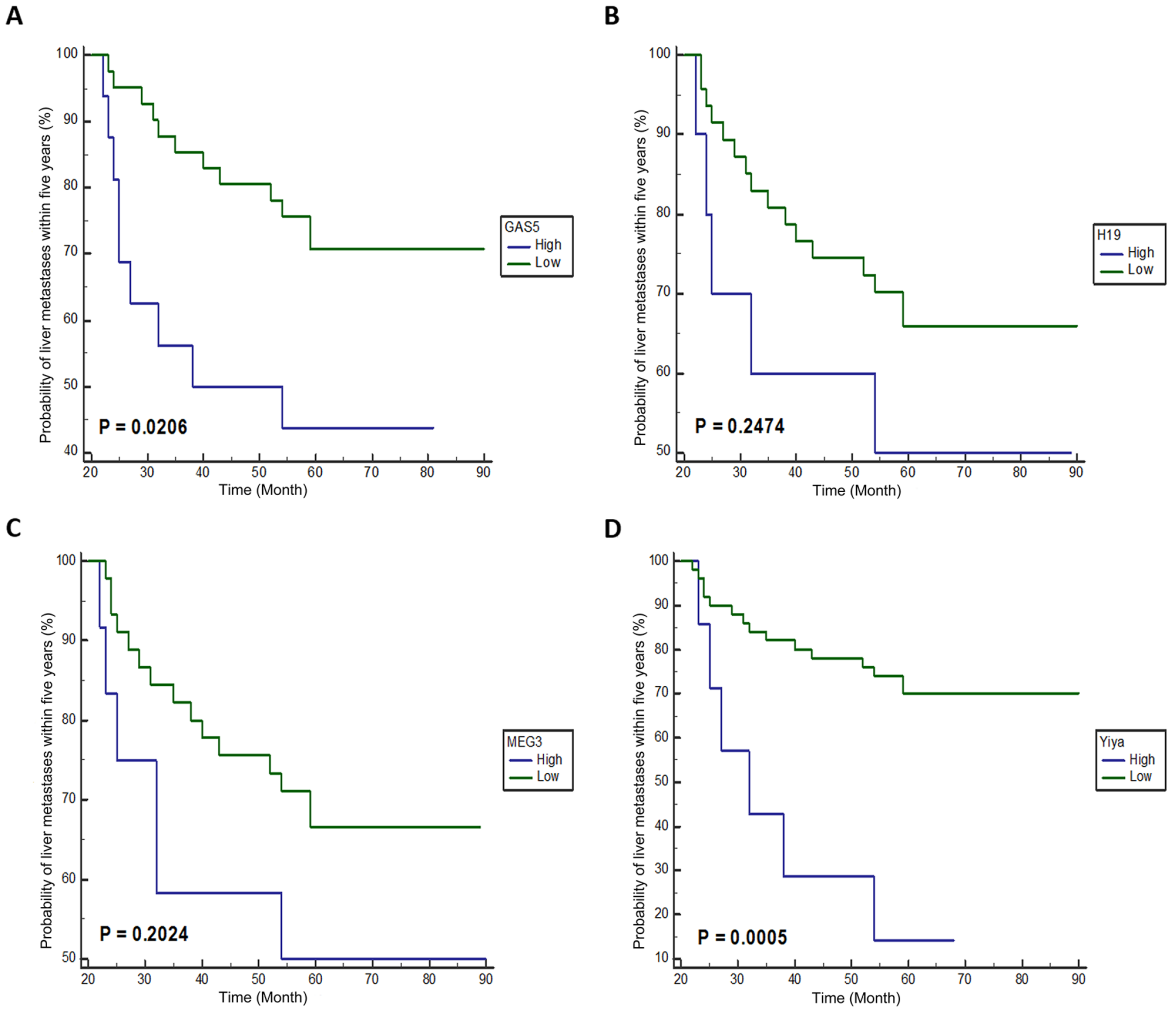
Kaplan-Meier curves of liver metastases on four candidate lncRNAs in patients with stage I/II CRC **A-D.** Kaplan-Meier curve of GAS5, H19, MEG3 and Yiya, respectively. High, high expression level ≥ mean 2^ΔCq^; Low, low expression level < mean 2^ΔCq^.

Figure 1 shows the Kaplan-Meier curves of liver metastases for 57 patients with early stage CRC classified according to lncRNAs expression levels. Remarkably, patients with high GAS5 or Yiya expression level had a high risk of liver metastases (p = 0.0206 and 0.0005 for GAS5 and Yiya, respectively). However, no significant correlation was found between the risk of liver metastases and the expression levels of H19 and MEG3.

In the univariate analysis, three other clinicopathological characteristics (age at diagnosis, depth and histologic grade of primary tumor, Table 4) were found significantly associated with colorectal liver metastases. Among all these factors, Cox proportional-hazards regression analysis showed that the expression level of Yiya was an independent indicator of liver metastases for early stage patients (relative risk = 10.7; p < 0.0001).

**Table 4:** Candidate lncRNAs and clinicopathological factors for predicting liver metastases in patients with stage I/II CRC

Factors	Subset	Univariate		Multivariate	
		P value	Relative risk	95% CI	P value
Age (year)	≤ 50 / > 50	0.0399	0.3	0.10 - 0.86	0.0257
Gender	Male / Female	0.3334	-	-	-
Organ	Colon / Rectum / C&R	0.1455	-	-	-
Depth^a^	T1 / T2 / T3 / T4	0.0137	2.2	1.16 - 4.29	0.0173
LVI	Positive / Negative	0.6198	-	-	-
Histologic grade	I / II / III	0.0317	2.6	0.95 - 7.24	0.0644
GAS5	High / Low	0.0206	3.3	0.77 - 14.55	0.1105
H19	High / Low	0.2474	-	-	-
MEG3	High / Low	0.2024	-	-	-
Yiya	High / Low	0.0005	10.7	3.40 - 33.52	0.0001

a According to Tumor-Node-Metastasis (TNM) Classification of Colorectal Carcinoma

High, high expression level ≥ mean 2^ΔCq^

Low, low expression level < mean 2^ΔCq^

CRC, colorectal cancer

C, colon; R, rectum; LVI, lymphatic vessel invasion; CI, confidence interval

## DISCUSSION

Liver metastasis is the primary cause of death for CRC patients [2]. A favorable prognosis should be expected for early stage individuals without lymph node and distant metastases. Still, 26% - 40% of CRC patients in stage I and II develop distant metastases and finally died of it within five years after surgery [3, 4]. Therefore, finding out predictive biomarkers of live metastases is meaningful to improve the prognosis and reduce the mortality of CRC patients.

Colorectal carcinomas tend to be enriched for the subclones that are adept at survival, growth, invasion and metastasis during progression [29]. Thus, compared with primary CRC, the metastatic deposits theoretically contain higher proportion of subclones with strong invasion and metastases ability. By comparing the expression levels of 15 lncRNAs between stage IV CRC and corresponding liver metastases, we identified four potential biomarkers, which are GAS5, H19, MEG3 and Yiya. We further validated that the high expression level of GAS5 or Yiya was highly correlated with the poor prognosis of early stage CRC patients in a cohort of 57 stage I/II samples. Finally, Yiya was proved to be an independent prognostic biomarker of colorectal liver metastases.

So far, investigations on prognostic biomarkers for CRC liver metastases have been reported in previous studies. MicroRNA-214 was identified as a negative regulator of colorectal liver metastases by regulating fibroblast growth factor receptor 1 (FGFR1) expression [30]. Serum microRNA-29a expression was reported to be higher in CRC with liver metastases than that without liver metastases, which has strong potential as a novel noninvasive biomarker for early detection of colorectal liver metastases [31]. With regard to lncRNA, high expression level of HOTAIR was revealed to be correlated with the presence of liver metastasis in CRC [32].

To our best knowledge, we are the first to report the prognostic values of GAS5 and Yiya in early prediction of colorectal liver metastases. Previously, the well-documented GAS5 was generally considered as a tumor suppressor. Down-regulation of GAS5 was found in multiple cancers, including CRC, breast cancer and hepatocellular carcinoma [20, 33, 34]. GAS5 expression was also suggested to be an indicator of overall survival in CRC and hepatocellular carcinoma [33, 34]. However, how GAS5 was involved in colorectal liver metastases has not been fully understood. Little is known about Yiya as it was discovered recently. Identified in a cancer susceptibility region, Yiya was found to be overexpressed in breast, hepatocellular, ovary, and esophageal cancers [15]. Interestingly, the study also suggested that there located a transcription factor Prospero-related homeobox 1 (PROX1) downstream of Yiya, which might be involved in cancer metastases.

Further functional studies of GAS5 and Yiya will enrich our knowledge to understand the underlying mechanisms of colorectal liver metastases. On the other hand, as colorectal liver metastases generally depend on portal drainage, GAS5 and Yiya have a good chance to be correlated with hematogenous spread. As such, the identification of these two lncRNAs would confer great benefit on patients with other types of cancers which tend to metastasize via hematogenous pathway.

In conclusion, our study reveals that GAS5 and Yiya are novel prognostic biomarkers to predict the risk of liver metastases for early stage CRC patients. These two lncRNAs have considerable clinical values in the early prediction and timely clinical intervention of CRC liver metastases.

## MATERIALS AND METHODS

### Ethics statement

Investigation has been conducted in accordance with the ethical standards and according to the Declaration of Helsinki and according to national and international guidelines and has been approved by the Institutional Review Board of Shanghai Medical College in Fudan University, with written informed consent obtained from all patients.

### Clinical specimens and study design

FFPE resection specimens of 51 stage IV colorectal carcinomas with matched liver metastases and 57 stage I/II colorectal carcinomas were collected from Zhongshan and Huashan Hospitals in Fudan University between March 2006 and July 2013. Routine histological classification according to the WHO criteria [35] was used to screen CRC samples. All cases were diagnosed by two pathologists and independently reviewed by an expert CRC pathologist. Patients who received preoperative radiotherapy or chemotherapy were excluded. Furthermore, stage I/II CRC patients who presented liver metastases within 12 months after surgery were also excluded. The clinical characteristics of patients and tumors in the study are presented in Table 2.

**Figure 2:**
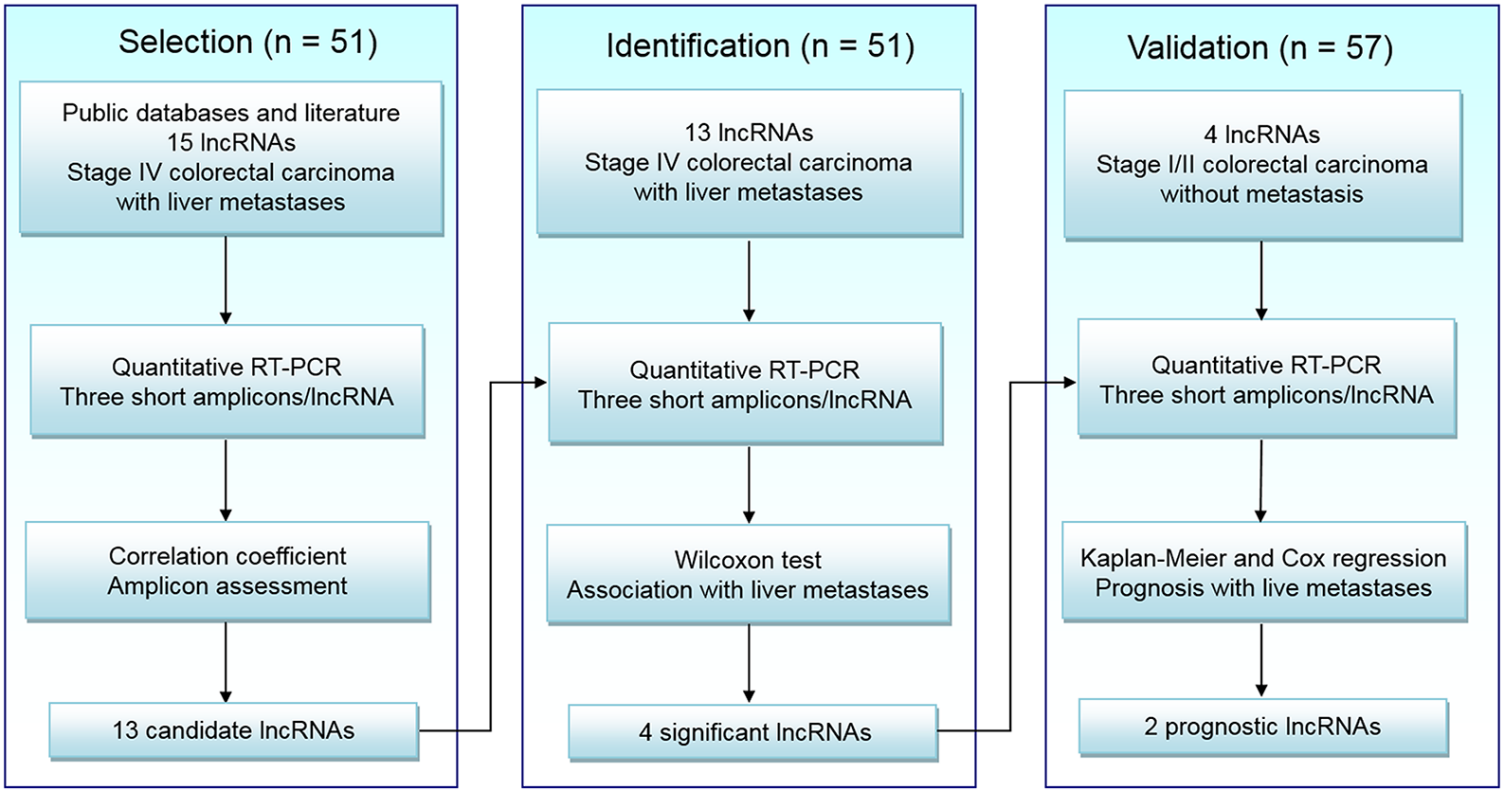
Study design LncRNA, long non-coding RNA; RT-PCR, reverse transcriptase polymerase chain reaction.

Our experiments mainly consisted of three different phases (Figure 2). In Selection phase, 15 lncRNAs associated with at least two types of cancer were collected from public databases and literature (Supplementary Table S2). Quantitative RT-PCR with three non-overlapping short amplicons [28] for each RNA was performed in 51 pairs of FFPE surgical tissues from stage IV CRC and matched liver metastases. Thirteen lncRNAs passing the quality control (Supplementary Table S1) were chosen for further analysis.

In Identification phase, quantitative RT-PCR was performed on the chosen 13 lncRNAs in the same 51 pairs of specimens. Four candidate lncRNAs were found correlated with colorectal liver metastases.

In Validation phase, based on follow-up data, an independent cohort of 57 stage I/II CRC patients was divided into two groups: 21 patients present liver metastases within five years and 36 do not. Quantitative RT-PCR was performed between two groups to assess the prognostic value of four candidate lncRNAs on colorectal liver metastases.

### Macrodissection

Macrodissection was necessary as described in our previous studies [28, 36, 37]. Hematoxylin-Eosin (H&E)-stained sections for each FFPE tissue block were prepared and reviewed by two expert CRC pathologists. If the proportion of neoplastic cells was larger than 75%, the corresponding sample was deemed suitable for experiments without purification.

If, however, the proportion is less than 75%, neoplastic cells area would be marked on the H&E sections as reference. The same area of corresponding sample was isolated with block trimming method or target tissue dissection method depending on the distribution of marked area.

### RNA isolation

Total RNA of manually macrodissected FFPE samples was isolated using RecoverAll Total Nucleic Acid Isolation Kit as instructed (Ambion, Austin, Texas, USA). DNase digestion was performed on the nucleic acid samples to eliminate the genomic DNA contamination before the final extraction of RNA. The concentration of RNA was measured by the NanoDrop 2000 Spectrophotometers (Thermo Fisher Scientific, Waltham, MA, USA). RNA samples were removed from this study, if the OD 260/280 ratio was less than 1.8.

### Quantitative RT-PCR

Three non-overlapping short amplicons (~ 60bp) were designed for each of 15 lncRNAs and *β-actin* gene in accordance with the general principles. The specificity of primer sets was assured by BLAST using the human genomic plus transcript database (Human G+T) and verified via melting curves obtained from quantitative RT-PCR. The expression of *β-actin* mRNA was used as an endogenous control. The primers were synthetized and purified by Sangon Biotech (Shanghai, China). Supplementary Table S3 presents the primer sequences and the amplification efficiency.

Short amplicons are more efficient and more sensitive than long amplicons in quantitative RT-PCR. And three of such short amplicons can address the problem that random fragmentation of lncRNA to different extent in different tissue types, which gives the assurance of quantification accuracy and reliability [28].

Following the guidelines on the minimal information for publication of quantitative real-time PCR experiments (MIQE) [38], quantitative RT-PCR was performed on 7900HT Fast Real-Time PCR System (Applied Biosystems) with High Capacity cDNA Reverse Transcription Kit (Invitrogen, Foster City, California, USA) and Power SYBR Green PCR Master Mix (Applied Biosystems, Warrington, UK). For reverse transcription, 500 ng of total RNA sample was reverse transcripted into 50 μl of cDNA solution with random primers. For real-time PCR, 6 μl of the cDNA solution was amplified with 16 μl 2x SYBR Green PCR Master mix and 2 μl target-specific primers (5 μM/L) in a final volume of 32 μl. All assays were carried out in triplicate. The Cq values were determined during 40 cycles of amplifications.

### Statistical analysis

The delta Cq (ΔCq) value was used to represent the expression level of lncRNA in quantitative RT-PCR. For each amplicon designed, the ΔCq value was normalized using the equation: ΔCq = Cq (target) – Cq (β-actin). The mean normalized ΔCq value of three short amplicons was also calculated.

Spearman rank correlation coefficient was used to measure the correlation of three non-overlapping short amplicons. The correlation was considered to be strong when the coefficient was between 0.70 and 1.00. The expression levels of lncRNA between stage IV CRC specimens and matched liver metastases were compared by Wilcoxon test for paired samples. Mann-Whitney unpaired test was used to evaluate the difference of lncRNA expression levels between stage I/II CRC patients presenting liver metastases within five years or not. Receiver operating characteristic (ROC) curve analysis was performed to assess the predictive performance of target lncRNAs. The area under the curve (AUC) with 95% confidence interval (CI) was used as an accuracy index of the prediction. The average expression level of each lncRNA was used as the cutoff. Low group of lncRNA expression was classified as values below the corresponding cutoff, while high group was classified as values at or above the corresponding cutoff. Kaplan-Meier methods are performed between low and high groups of four lncRNAs to analyze the correlation between lncRNAs expression levels and prognosis on liver metastases of stage I/II CRC patients. A Cox proportional hazard model was used for multivariate analysis.

MedCalc software (version 10.4.7.0; MedCalc, Mariakerke, Belgium) was used for statistical analysis. All p values were two-tailed and the difference was considered to be significant when p value was less than 0.0500.

## SUPPLEMENTARY MATERIALS FIGURES AND TABLES








