# Supplementary material for: Long non-coding RNAs: novel prognostic biomarkers for liver metastases in patients with early stage colorectal cancer
Source: Oncotarget. 2016 Jul 6;7(31):50428–36. doi: 10.18632/oncotarget.10416 (PMC5226593; doi:10.18632/oncotarget.10416)
Supplement: Supplementary file 1 [file oncotarget-07-50428-s001.pdf]

Long non-coding RNAs: novel prognostic biomarkers for liver metastases in patients with early stage colorectal cancer

SUPPLEMENTARY FIGURES AND TABLES

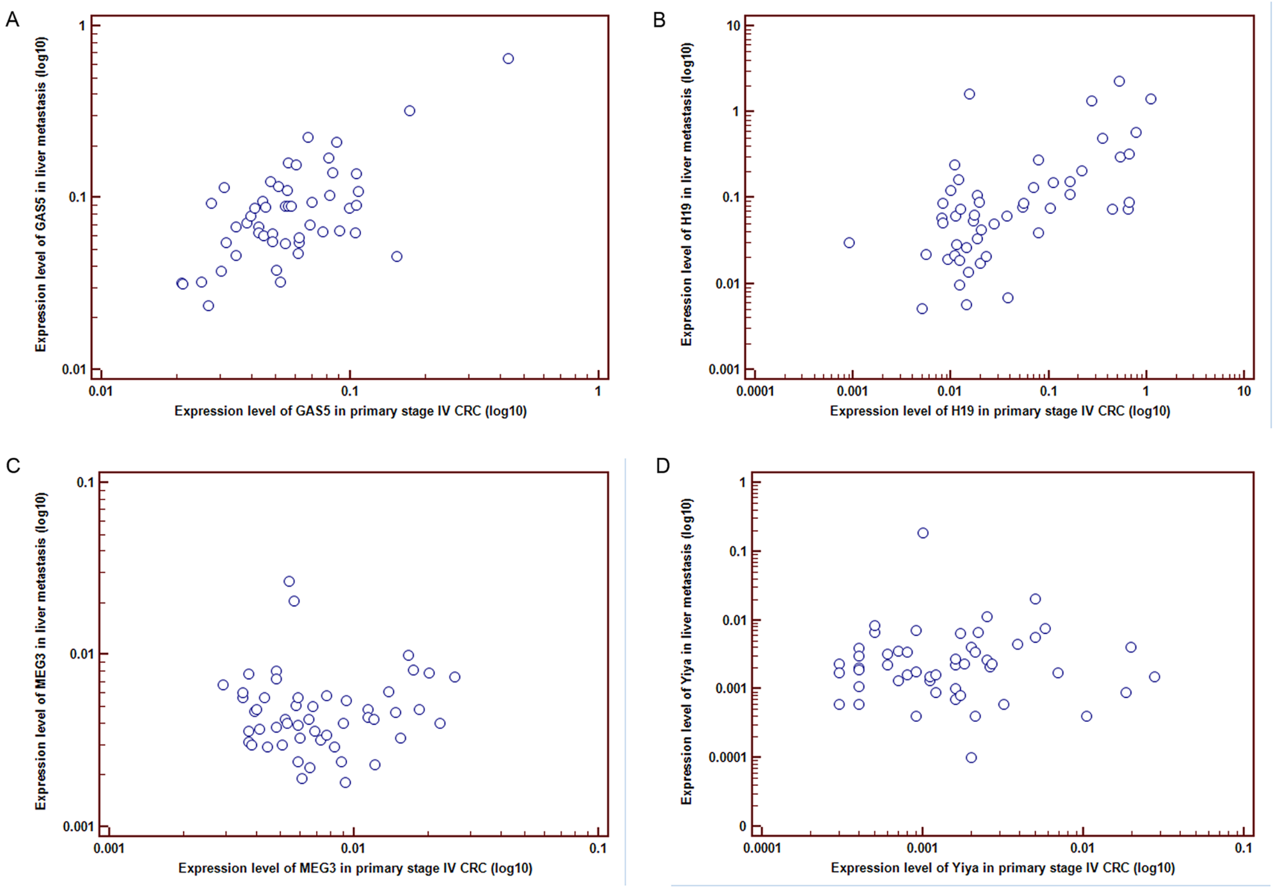

Supplementary Figure S1: Scatter-plots indicating the differential expressions of four candidate lncRNAs between primary CRC and liver metastases. A-D. Scatter-plots of GAS5, H19, MEG3 and Yiya, respectively.

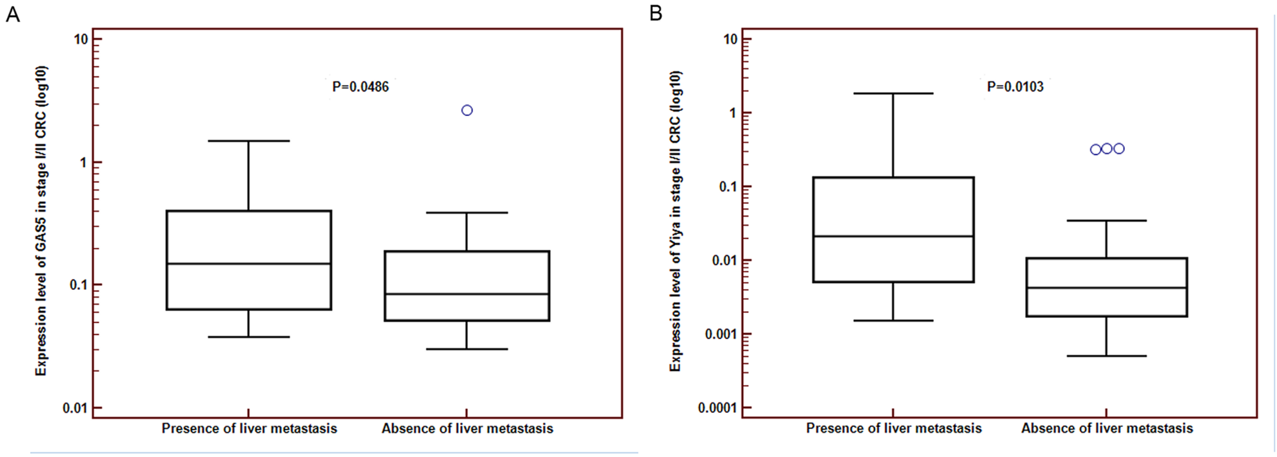

Supplementary Figure S2: Box-plots indicating the differential expressions of GAS5 and Yiya in stage I/II CRC with and without liver metastases. A-B. Box-plots of GAS5 and Yiya, respectively.

Supplementary Table S1: Quality assessment of short amplicons in stage IV colorectal carcinoma with liver metastases.

See Supplementary File 1

Supplementary Table S2: The expression levels of collected lncRNAs in different tumor types.

See Supplementary File 2

Supplementary Table S3: Primer sequence and amplification efficiency of short amplicons.

See Supplementary File 3
