# Supplementary material for: Long non-coding RNAs: novel prognostic biomarkers for liver metastases in patients with early stage colorectal cancer
Source: Oncotarget. 2016 Jul 6;7(31):50428–36. doi: 10.18632/oncotarget.10416 (PMC5226593; doi:10.18632/oncotarget.10416)
Supplement: Supplementary file 2 [file oncotarget-07-50428-s002.doc]

**Supplementary Table S1** Quality assessment of short amplicons in stage IV colorectal carcinoma with liver metastases

|  | **Primer set** | **Colorectal carcinoma** | |  | **Liver metastases** | |
| --- | --- | --- | --- | --- | --- | --- |
|  |  | Mean Cq value | Correlation coefficient |  | Mean Cq value | Correlation coefficient |
| β-actin | a | 24 | 0.94 |  | 23 | 0.95 |
|  | b | 24 | 0.90 |  | 23 | 0.90 |
|  | c | 23 | 0.88 |  | 22 | 0.90 |
|  |  |  |  |  |  |  |
| CCAT1 | a | 32 | 0.92 |  | 32 | 0.96 |
|  | b | 29 | 0.97 |  | 28 | 0.97 |
|  | c | 30 | 0.97 |  | 29 | 0.98 |
|  |  |  |  |  |  |  |
| GAS5 | a | 27 | 0.92 |  | 27 | 0.96 |
|  | b | 28 | 0.86 |  | 27 | 0.92 |
|  | c | 27 | 0.95 |  | 26 | 0.96 |
|  |  |  |  |  |  |  |
| H19 | a | 29 | 0.94 |  | 28 | 0.95 |
|  | b | 28 | 0.96 |  | 27 | 0.94 |
|  | c | 28 | 0.98 |  | 27 | 0.98 |
|  |  |  |  |  |  |  |
|  | a | 33 | 0.63 |  | 34 | 0.82 |
| *HOTAIR | b | 33 | 0.91 |  | 34 | 0.80 |
|  | c | 32 | 0.91 |  | 33 | 0.85 |
|  |  |  |  |  |  |  |
| IGF2-AS | a | 33 | 0.80 |  | 33 | 0.76 |
|  | b | 34 | 0.75 |  | 33 | 0.74 |
|  | c | 34 | 0.82 |  | 33 | 0.81 |
|  |  |  |  |  |  |  |
| lncRNA-LET | a | 31 | 0.94 |  | 31 | 0.96 |
|  | b | 31 | 0.87 |  | 31 | 0.89 |
|  | c | 32 | 0.94 |  | 31 | 0.95 |
|  |  |  |  |  |  |  |
| MALAT1 | a | 23 | 0.94 |  | 23 | 0.94 |
|  | b | 31 | 0.89 |  | 31 | 0.93 |
|  | c | 22 | 0.91 |  | 22 | 0.94 |
|  |  |  |  |  |  |  |
| MEG3 | a | 31 | 0.96 |  | 31 | 0.97 |
|  | b | 30 | 0.87 |  | 31 | 0.95 |
|  | c | 30 | 0.93 |  | 31 | 0.96 |
|  |  |  |  |  |  |  |
| MIR17HG | a | 30 | 0.81 |  | 30 | 0.89 |
|  | b | 32 | 0.67 |  | 32 | 0.78 |
|  | c | 32 | 0.88 |  | 31 | 0.92 |
|  |  |  |  |  |  |  |
| p15AS | a | 33 | 0.65 |  | 33 | 0.70 |
|  | b | 32 | 0.86 |  | 33 | 0.82 |
|  | c | 33 | 0.70 |  | 33 | 0.70 |
|  |  |  |  |  |  |  |
| *PANDAR | a | 32 | 0.72 |  | 32 | 0.65 |
|  | b | 32 | 0.66 |  | 33 | 0.87 |
|  | c | 33 | 0.73 |  | 32 | 0.73 |
|  |  |  |  |  |  |  |
| PVT1 | a | 29 | 0.97 |  | 29 | 0.97 |
|  | b | 32 | 0.90 |  | 31 | 0.95 |
|  | c | 28 | 0.93 |  | 27 | 0.95 |
|  |  |  |  |  |  |  |
| UCA1 | a | 30 | 0.95 |  | 30 | 0.96 |
|  | b | 32 | 0.90 |  | 31 | 0.90 |
|  | c | 31 | 0.97 |  | 31 | 0.98 |
|  |  |  |  |  |  |  |
| XIST | a | 30 | 0.96 |  | 30 | 0.94 |
|  | b | 31 | 0.99 |  | 30 | 0.96 |
|  | c | 30 | 0.98 |  | 30 | 0.98 |
|  |  |  |  |  |  |  |
| Yiya | a | 34 | 0.88 |  | 33 | 0.87 |
|  | b | 34 | 0.84 |  | 33 | 0.82 |
|  | c | 32 | 0.90 |  | 31 | 0.91 |
|  |  |  |  |  |  |  |

*Two lncRNAs (HOTAIR and PANDAR) were excluded for further evaluation due to the low expression level with Cq values > 35 cycles in more than 20% of the tested samples.
