# Supplementary material for: Long non-coding RNAs: novel prognostic biomarkers for liver metastases in patients with early stage colorectal cancer
Source: Oncotarget. 2016 Jul 6;7(31):50428–36. doi: 10.18632/oncotarget.10416 (PMC5226593; doi:10.18632/oncotarget.10416)
Supplement: Supplementary file 3 [file oncotarget-07-50428-s003.doc]

**Supplementary Table S2** The expression levels of collected lncRNAs in different tumor types

| **lncRNA** | **Position/Annotation** | **Cancer** | **Expression** | **Reference** |
| --- | --- | --- | --- | --- |
| CCAT1 | ENSG00000247844.1 | CRC | ↑Patient tissue |  |
|  |  | gastric carcinoma | ↑Patient tissue |  |
|  |  | gallbladder cancer | ↑Patient tissue |  |
|  |  | HCC | ↑Patient tissue |  |
| GAS5 | ENSG00000234741.3 | breast cancer | ↓Patient tissue | [20] |
|  |  | CRC | ↓Patient tissue | [34] |
|  |  | gastric carcinoma | ↓Patient tissue |  |
|  |  | HCC | ↓Patient tissue | [33] |
|  |  | NSCLC | ↓Patient tissue |  |
|  |  | prostate cancer | ↓Cell lines |  |
| H19 | ENSG00000130600.11 | bladder cancer | ↑Patient tissue |  |
|  |  | CRC | ↑Patient tissue/cell lines |  |
|  |  | gastric cancer | ↑Patient tissue/cell lines |  |
|  |  | HCC | ↑Patient tissue |  |
| HOTAIR | ENSG00000228630.1 | breast cancer | ↑Patient tissue | [8] |
|  |  | CRC | ↑Patient tissue |  |
|  |  | HCC | ↑Patient tissue |  |
|  |  | NSCLC | ↑Patient tissue |  |
|  |  | pancreatic cancer | ↑Patient tissue |  |
| IGF2-AS | ENSG00000099869.6 | HCC | N/A | [17] |
|  |  | Wilms' tumor | ↑Patient tissue |  |
| lncRNA-LET | ENSG00000281183 | CRC | ↓Patient tissue |  |
|  |  | HCC | ↓Patient tissue |  |
|  |  | SQ | ↓Patient tissue |  |
| MALAT1 | ENSG00000251562.3 | bladder cancer | ↑Patient tissue |  |
|  |  | CRC | ↑Patient tissue |  |
|  |  | HCC | ↑Patient tissue/cell lines |  |
|  |  | NSCLC | ↑Cell lines | [11], |
| MEG3 | ENSG00000214548.10 | bladder cancer | ↓Patient tissue |  |
|  |  | CRC | ↓Patient tissue |  |
|  |  | gastric cancer | ↓Patient tissue |  |
|  |  | HCC | ↓Patient tissue/cell lines |  |
|  |  | NSCLC | ↓Patient tissue |  |
| MIR17HG | ENSG00000215417.6 | breast cancer | N/A |  |
|  |  | lymphoma | N/A |  |
| p15AS | ENSG00000240498.2 | gastric cancer | ↑Patient tissue |  |
|  |  | prostate cancer | ↑Patient tissue |  |
| PANDAR | ENSG00000281450 | breast cancer | ↑Patient tissue/cell lines |  |
|  |  | gastric cancer | ↑Patient tissue |  |
|  |  | HCC | ↑Patient tissue/cell lines |  |
|  |  | NSCLC | ↓Patient tissue |  |
| PVT1 | ENSG00000249859.3 | breast cancer | ↑Cell lines |  |
|  |  | cholangiocarcinoma | ↑biliary brushings |  |
|  |  | CRC | ↑Patient tissue/cell lines |  |
|  |  | HCC | ↑Patient tissue |  |
|  |  | ovarian cancer | ↑Cell lines |  |
|  |  | pancreatic cancer | ↑Cell lines |  |
| UCA1 | ENSG00000214049.6 | bladder cancer | ↑Patient tissue | [25] |
|  |  | breast cancer | ↑Cell lines |  |
|  |  | CRC | ↑Patient tissue/cell lines |  |
|  |  | gastric cancer | ↑Patient tissue/cell lines |  |
|  |  | HCC | ↑Patient tissue |  |
| XIST | ENSG00000229807.5 | breast cancer | ↓Cell lines |  |
|  |  |  | ↓Brain metastatic breast cancer tissue/cell lines |  |
|  |  | cervical cancer | ↓Cell lines |  |
|  |  | ovarian cancer | ↓Cell lines |  |
| Yiya | LINC00538* | breast cancer | ↑Patient tissue/cell lines | [15] |
|  |  | HCC | ↑Patient tissue/cell lines | [15] |

*: Provided by HUGO Gene Nomenclature Committee (HGNC);

↑: up-regulated expression; ↓: down-regulated expression

CRC: colorectal cancer; HCC: hepatocellular carcinoma; NSCLC: non-small cell lung cancer; SQ: squamous-cell lung cancer

**SUPPLEMENTARY REFERENCES**

S1. Ye Z, Zhou M, Tian B, Wu B and Li J. Expression of lncRNA-CCAT1, E-cadherin and N-cadherin in colorectal cancer and its clinical significance. International Journal of Clinical and Experimental Medicine. 2015; 8: 3707-15.

S2. Yang F, Xue X, Bi J, Zheng L, Zhi K, Gu Y and Fang G. Long noncoding RNA CCAT1, which could be activated by c-Myc, promotes the progression of gastric carcinoma. J Cancer Res Clin Oncol. 2013; 139: 437-45.

S3. Ma M, Chu B, Zhang Y, Weng M, Qin Y, Gong W and Quan Z. Long non-coding RNA CCAT1 promotes gallbladder cancer development via negative modulation of miRNA-218-5p. Cell death & disease. 2015; 6: e1583.

S4. Deng L, Yang S-B, Xu F-F and Zhang J-H. Long noncoding RNA CCAT1 promotes hepatocellular carcinoma progression by functioning as let-7 sponge. Journal of Experimental & Clinical Cancer Research. 2015; 34: 1.

S5. Sun M, Jin F-y, Xia R, Kong R, Li J-h, Xu T-p, Liu Y-w, Zhang E-b, Liu X-h and De W. Decreased expression of long noncoding RNA GAS5 indicates a poor prognosis and promotes cell proliferation in gastric cancer. BMC Cancer. 2014; 14: 1-12.

S6. Shi X, Sun M, Liu H, Yao Y, Kong R, Chen F and Song Y. A critical role for the long non-coding RNA GAS5 in proliferation and apoptosis in non-small-cell lung cancer. Molecular Carcinogenesis. 2015; 54: E1-E12.

S7. Pickard MR, Mourtada-Maarabouni M and Williams GT. Long non-coding RNA GAS5 regulates apoptosis in prostate cancer cell lines. Biochimica et Biophysica Acta (BBA) - Molecular Basis of Disease. 2013; 1832: 1613-23.

S8. Luo M, Li Z, Wang W, Zeng Y, Liu Z and Qiu J. Long non-coding RNA H19 increases bladder cancer metastasis by associating with EZH2 and inhibiting E-cadherin expression. Cancer Letters. 333: 213-21.

S9. Tsang WP, Ng EK, Ng SS, Jin H, Yu J, Sung JJ and Kwok TT. Oncofetal H19-derived miR-675 regulates tumor suppressor RB in human colorectal cancer. Carcinogenesis. 2010; 31: 350-8.

S10. Yang F, Bi J, Xue X, Zheng L, Zhi K, Hua J and Fang G. Up‐regulated long non‐coding RNA H19 contributes to proliferation of gastric cancer cells. FEBS Journal. 2012; 279: 3159-65.

S11. Ariel I, Miao HQ, Ji XR, Schneider T, Roll D, de Groot N, Hochberg A and Ayesh S. Imprinted H19 oncofetal RNA is a candidate tumour marker for hepatocellular carcinoma. Molecular Pathology. 1998; 51: 21-5.

S12. Kogo R, Shimamura T, Mimori K, Kawahara K, Imoto S, Sudo T, Tanaka F, Shibata K, Suzuki A, Komune S, Miyano S and Mori M. Long noncoding RNA HOTAIR regulates polycomb-dependent chromatin modification and is associated with poor prognosis in colorectal cancers. Cancer Res. 2011; 71: 6320-6.

S13. Geng Y, Xie S, Li Q, Ma J and Wang G. Large intervening non-coding RNA HOTAIR is associated with hepatocellular carcinoma progression. Journal of International Medical Research. 2011; 39: 2119-28.

S14. Nakagawa T, Endo H, Yokoyama M, Abe J, Tamai K, Tanaka N, Sato I, Takahashi S, Kondo T and Satoh K. Large noncoding RNA HOTAIR enhances aggressive biological behavior and is associated with short disease-free survival in human non-small cell lung cancer. Biochemical and biophysical research communications. 2013; 436: 319-24.

S15. Kim K, Jutooru I, Chadalapaka G, Johnson G, Frank J, Burghardt R, Kim S and Safe S. HOTAIR is a negative prognostic factor and exhibits pro-oncogenic activity in pancreatic cancer. Oncogene. 2013; 32: 1616-25.

S16. Okutsu T, Kuroiwa Y, Kagitani F, Kai M, Aisaka K, Tsutsumi O, Kaneko Y, Yokomori K, Surani MA, Kohda T, Kaneko-Ishino T and Ishino F. Expression and imprinting status of human PEG8/IGF2AS, a paternally expressed antisense transcript from the IGF2 locus, in Wilms' tumors. J Biochem. 2000; 127: 475-83.

S17. Yang F, Huo XS, Yuan SX, Zhang L, Zhou WP, Wang F and Sun SH. Repression of the long noncoding RNA-LET by histone deacetylase 3 contributes to hypoxia-mediated metastasis. Mol Cell. 2013; 49: 1083-96.

S18. Ying L, Chen Q, Wang Y, Zhou Z, Huang Y and Qiu F. Upregulated MALAT-1 contributes to bladder cancer cell migration by inducing epithelial-to-mesenchymal transition. Molecular BioSystems. 2012; 8: 2289-94.

S19. Zheng H-T, Shi D-B, Wang Y-W, Li X-X, Xu Y, Tripathi P, Gu W-L, Cai G-X and Cai S-J. High expression of lncRNA MALAT1 suggests a biomarker of poor prognosis in colorectal cancer. Int J Clin Exp Pathol. 2014; 7: 3174-81.

S20. Lai M-c, Yang Z, Zhou L, Zhu Q-q, Xie H-y, Zhang F, Wu L-m, Chen L-m and Zheng S-s. Long non-coding RNA MALAT-1 overexpression predicts tumor recurrence of hepatocellular carcinoma after liver transplantation. Medical Oncology. 2012; 29: 1810-6.

S21. Schmidt LH, Spieker T, Koschmieder S, Humberg J, Jungen D, Bulk E, Hascher A, Wittmer D, Marra A and Hillejan L. The long noncoding MALAT-1 RNA indicates a poor prognosis in non-small cell lung cancer and induces migration and tumor growth. Journal of Thoracic Oncology. 2011; 6: 1984-92.

S22. Ying L, Huang Y, Chen H, Wang Y, Xia L, Chen Y, Liu Y and Qiu F. Downregulated MEG3 activates autophagy and increases cell proliferation in bladder cancer. Molecular BioSystems. 2013; 9: 407-11.

S23. Yin D-d, Liu Z-j, Zhang E, Kong R, Zhang Z-h and Guo R-h. Decreased expression of long noncoding RNA MEG3 affects cell proliferation and predicts a poor prognosis in patients with colorectal cancer. Tumor Biology. 2015; 36: 4851-9.

S24. Sun M, Xia R, Jin F, Xu T, Liu Z, De W and Liu X. Downregulated long noncoding RNA MEG3 is associated with poor prognosis and promotes cell proliferation in gastric cancer. Tumor Biology. 2014; 35: 1065-73.

S25. Braconi C, Kogure T, Valeri N, Huang N, Nuovo G, Costinean S, Negrini M, Miotto E, Croce CM and Patel T. microRNA-29 can regulate expression of the long non-coding RNA gene MEG3 in hepatocellular cancer. Oncogene. 2011; 30: 4750-6.

S26. Lu K-h, Li W, Liu X-h, Sun M, Zhang M-l, Wu W-q, Xie W-p and Hou Y-y. Long non-coding RNA MEG3 inhibits NSCLC cells proliferation and induces apoptosis by affecting p53 expression. BMC cancer. 2013; 13: 461.

S27. Chacon-Cortes D, Smith RA, Lea RA, Youl PH and Griffiths LR. Association of microRNA 17–92 cluster host gene (MIR17HG) polymorphisms with breast cancer. Tumor Biology. 2015; 36: 5369-76.

S28. Ota A, Tagawa H, Karnan S, Tsuzuki S, Karpas A, Kira S, Yoshida Y and Seto M. Identification and characterization of a novel gene, C13orf25, as a target for 13q31-q32 amplification in malignant lymphoma. Cancer Res. 2004; 64: 3087-95.

S29. Zhang EB, Kong R, Yin DD, You LH, Sun M, Han L, Xu TP, Xia R, Yang JS, De W and Chen J. Long noncoding RNA ANRIL indicates a poor prognosis of gastric cancer and promotes tumor growth by epigenetically silencing of miR-99a/miR-449a. Oncotarget. 2014; 5: 2276-92.

S30. Yap KL, Li S, Muñoz-Cabello AM, Raguz S, Zeng L, Mujtaba S, Gil J, Walsh MJ and Zhou M-M. Molecular Interplay of the Non-coding RNA ANRIL and Methylated Histone H3 Lysine 27 by Polycomb CBX7 in Transcriptional Silencing of INK4a. Molecular cell. 2010; 38: 662-74.

S31. Sang Y, Tang J, Li S, Li L, Tang X, Cheng C, Luo Y, Qian X, Deng L-M, Liu L and Lv X-B. LncRNA PANDAR regulates the G1/S transition of breast cancer cells by suppressing p16INK4A expression. Scientific Reports. 2016; 6: 22366.

S32. Ma P, Xu T, Huang M and Shu Y. Increased expression of LncRNA PANDAR predicts a poor prognosis in gastric cancer. Biomedicine & Pharmacotherapy. 2016; 78: 172-6.

S33. Peng W and Fan H. Long non-coding RNA PANDAR correlates with poor prognosis and promotes tumorigenesis in hepatocellular carcinoma. Biomedicine & Pharmacotherapy. 2015; 72: 113-8.

S34. Han L, Zhang Eb, Yin Dd, Kong R, Xu Tp, Chen Wm, Xia R, Shu Yq and De W. Low expression of long noncoding RNA PANDAR predicts a poor prognosis of non-small cell lung cancer and affects cell apoptosis by regulating Bcl-2. Cell Death Dis. 2015; 6: e1665.

S35. Guan Y, Kuo W-L, Stilwell JL, Takano H, Lapuk AV, Fridlyand J, Mao J-H, Yu M, Miller MA, Santos JL, Kalloger SE, Carlson JW, Ginzinger DG, et al. Amplification of PVT1 Contributes to the Pathophysiology of Ovarian and Breast Cancer. Clinical Cancer Research. 2007; 13: 5745-55.

S36. Chapman MH, Tidswell R, Dooley JS, Sandanayake NS, Cerec V, Deheragoda M, Lee AJX, Swanton C, Andreola F and Pereira SP. Whole genome RNA expression profiling of endoscopic biliary brushings provides data suitable for biomarker discovery in cholangiocarcinoma. Journal of Hepatology. 56: 877-85.

S37. Takahashi Y, Sawada G, Kurashige J, Uchi R, Matsumura T, Ueo H, Takano Y, Eguchi H, Sudo T and Sugimachi K. Amplification of PVT-1 is involved in poor prognosis via apoptosis inhibition in colorectal cancers. British journal of cancer. 2014; 110: 164-71.

S38. Wang F, Yuan JH, Wang SB, Yang F, Yuan SX, Ye C, Yang N, Zhou WP, Li WL and Li W. Oncofetal long noncoding RNA PVT1 promotes proliferation and stem cell‐like property of hepatocellular carcinoma cells by stabilizing NOP2. Hepatology. 2014; 60: 1278-90.

S39. You L, Chang D, Du H-Z and Zhao Y-P. Genome-wide screen identifies PVT1 as a regulator of Gemcitabine sensitivity in human pancreatic cancer cells. Biochemical and Biophysical Research Communications. 2011; 407: 1-6.

S40. Huang J, Zhou N, Watabe K, Lu Z, Wu F, Xu M and Mo YY. Long non-coding RNA UCA1 promotes breast tumor growth by suppression of p27 (Kip1). Cell Death Dis. 2014; 5: e1008.

S41. Han Y, Yang Y-n, Yuan H-h, Zhang T-t, Sui H, Wei X-l, Liu L, Huang P, Zhang W-j and Bai Y-x. UCA1, a long non-coding RNA up-regulated in colorectal cancer influences cell proliferation, apoptosis and cell cycle distribution. Pathology-Journal of the RCPA. 2014; 46: 396-401.

S42. Zheng Q, Wu F, Dai WY, Zheng DC, Zheng C, Ye H, Zhou B, Chen JJ and Chen P. Aberrant expression of UCA1 in gastric cancer and its clinical significance. Clin Transl Oncol. 2015; 17: 640-6.

S43. Wang F, Ying HQ, He BS, Pan YQ, Deng QW, Sun HL, Chen J, Liu X and Wang SK. Upregulated lncRNA-UCA1 contributes to progression of hepatocellular carcinoma through inhibition of miR-216b and activation of FGFR1/ERK signaling pathway. Oncotarget. 2015; 6: 7899-917.

S44. Liu Y, Xing F, Wu K, Sharma S and Watabe K. Abstract B22: Loss of LncRNA XIST induces Epithelial to Mesenchymal Transition in Breast Cancer. Cancer Research. 2016; 76: B22.

S45. Kawakami T, Zhang C, Taniguchi T, Kim CJ, Okada Y, Sugihara H, Hattori T, Reeve AE, Ogawa O and Okamoto K. Characterization of loss-of-inactive X in Klinefelter syndrome and female-derived cancer cells. Oncogene. 2004; 23: 6163-9.
