# Supplementary material for: Long non-coding RNAs: novel prognostic biomarkers for liver metastases in patients with early stage colorectal cancer
Source: Oncotarget. 2016 Jul 6;7(31):50428–36. doi: 10.18632/oncotarget.10416 (PMC5226593; doi:10.18632/oncotarget.10416)
Supplement: Supplementary file 4 [file oncotarget-07-50428-s004.doc]

**Supplementary Table S3 Primer sequence and amplification efficiency of short amplicons**

|  | **Accession** | **Size (bp)** | **Primer set** | **Sequence (5' to 3')** | **Amplicon (bp)** | **Efficient** | **Correlation coefficient** |
| --- | --- | --- | --- | --- | --- | --- | --- |
| **Endogenous control** | |  |  |  |  |  |  |
| β-actin | NM_001101.3 | 1,852 | a | GCATGGGTCAGAAGGATTCC | 58 | 100.8% | 0.9996 |
|  |  |  |  | AGGATGCCTCTCTTGCTCTG |  |  |  |
|  |  |  | b | GCGTGACATTAAGGAGAAGC | 53 | 99.8% | 0.9961 |
|  |  |  |  | TCTCTTGCTCGAAGTCCAGG |  |  |  |
|  |  |  | c | AAGGTGACAGCAGTCGGTTG | 54 | 97.0% | 0.9991 |
|  |  |  |  | CGGCCACATTGTGAACTTTG |  |  |  |
| **lncRNA** |  |  |  |  |  |  |  |
| CCAT1 | NR_108049.1 | 2,795 | a | TTGAAGTTGCACTGACCTGG | 59 | 103.6% | 0.9504 |
|  |  |  |  | TTAGCCATACAGAGCCAACC |  |  |  |
|  |  |  | b | GATTCTGTGTAAGCACTGGC | 51 | 77.8% | 0.9896 |
|  |  |  |  | AAAGAGAGGAGAAGGCATTC |  |  |  |
|  |  |  | c | GTCTTCCACAAAACTGGTCC | 53 | 66.7% | 0.9551 |
|  |  |  |  | TAAAGCAGTGGTCCCAAGCC |  |  |  |
|  |  |  |  |  |  |  |  |
| GAS5 | NR_002578.2 | 651 | a | GCTCTGGATAGCACCTTATG | 51 | 102.6% | 0.9972 |
|  |  |  |  | ATTCTCATCCTTCCTTGGGG |  |  |  |
|  |  |  | b | TGAAGAAATGCAGGCAGACC | 66 | 125.4% | 0.9893 |
|  |  |  |  | CATGCTTGCTTGTTGTGGTC |  |  |  |
|  |  |  | c | TCAAGTGATCCTCAGCCTCC | 62 | 98.9% | 0.9953 |
|  |  |  |  | TTGTGCCATGAGACTCCATC |  |  |  |
|  |  |  |  |  |  |  |  |
| H19 | NR_002196.1 | 2,322 | a | GTCATTTGCACTGGTTGGAG | 52 | 104.6% | 0.9988 |
|  |  |  |  | ACTCGTACTGAGACTCAAGG |  |  |  |
|  |  |  | b | AGCTAGGGCTGGAAAGAAGG | 57 | 96.2% | 0.9971 |
|  |  |  |  | TGTAACCAAAAGTGACCGGG |  |  |  |
|  |  |  | c | GCTGCACTTTACAACCACTG | 55 | 95.4% | 0.9966 |
|  |  |  |  | TCACCTTCCAGAGCCGATTC |  |  |  |
|  |  |  |  |  |  |  |  |
| HOTAIR | 3 transcripts | 2,337-2,370 | a | AAGAGAGCGCCAGACGAAGG | 55 | 114.5% | 0.9791 |
|  |  |  |  | CCCTTGCCTGCATTTCTCTG |  |  |  |
|  |  |  | b | TGGGACCAATTTTAGGAGGC | 56 | 104.8% | 0.9620 |
|  |  |  |  | GGAAGCATTTTCTGACACTG |  |  |  |
|  |  |  | c | GCTAAATAGACTCAGGACTG | 52 | 103.6% | 0.9558 |
|  |  |  |  | ACCACACACACACAACCTAC |  |  |  |
|  |  |  |  |  |  |  |  |
| IGF2-AS | 2 transcripts | 2,091-2,881 | a | AACCCTCCACACCAGACAGC | 55 | 100.6% | 0.8639 |
|  |  |  |  | CTTTGGGCAGATTGAGCAGC |  |  |  |
|  |  |  | b | TGGAAGGAGATAAGGAGGGG | 56 | 128.3% | 0.8270 |
|  |  |  |  | AAGCAGAGCTGTGTGTCCAG |  |  |  |
|  |  |  | c | TCCTCCACCTCCAAACACCC | 72 | 95.4% | 0.9485 |
|  |  |  |  | GTGTGTCCAGTGGCTTTTGC |  |  |  |
|  |  |  |  |  |  |  |  |
| lncRNA-LET | NR_103844.1 | 2,606 | a | GAGAGTCTGATGTATCCACC | 52 | 96.3% | 0.9897 |
|  |  |  |  | CCGCCTTTGCTATCTCTGTC |  |  |  |
|  |  |  | b | CAGAAAGAATGTGGCCCCAG | 53 | 100.6% | 0.9797 |
|  |  |  |  | CCTCCCAGAGAGTCTAAAGC |  |  |  |
|  |  |  | c | TAAAAGAGATGACGGCAGGG | 56 | 103.2% | 0.9813 |
|  |  |  |  | AAGAGACTAGAGCAAGGAGG |  |  |  |
|  |  |  |  |  |  |  |  |
| MALAT1 | NR_002819.2 | 8,708 | a | GTAGACTGGAGAAGATAGGC | 54 | 105.9% | 0.9991 |
|  |  |  |  | ATTCCCACCCAAAAGCCCTC |  |  |  |
|  |  |  | b | TCCAAGAGTGGGTTTTCACG | 68 | 106.50% | 0.9512 |
|  |  |  |  | TCGAGAAATCGGAGCAGCAC |  |  |  |
|  |  |  | c | CCAGGAACCAGTGTTTGATG | 65 | 95.21% | 0.9986 |
|  |  |  |  | TCACCACGAACTGCTGCTTG |  |  |  |
|  |  |  |  |  |  |  |  |
| MEG3 | 16 transcripts | 1,506-9,701 | a | CTCCTTCACCTACCTCACAG | 57 | 100.5% | 0.9879 |
|  |  |  |  | GCTTTGGAACCGCATCACAG |  |  |  |
|  |  |  | b | CCCACCAACATACAAAGCAG | 57 | 96.0% | 0.9823 |
|  |  |  |  | ATCCTTTGCCATCCTGGTCC |  |  |  |
|  |  |  | c | CTGGCATAGAGGAGGTGATC | 59 | 91.6% | 0.9946 |
|  |  |  |  | AGGGACTGACCTGTCAAACC |  |  |  |
|  |  |  |  |  |  |  |  |
| MIR17HG | 2 transcripts | 923-5,081 | a | AGACCTGTCTAACTACAAGC | 62 | 99.3% | 0.9774 |
|  |  |  |  | GAGTCAGTGTGTCTTCAAAC |  |  |  |
|  |  |  | b | GCAGTAAAGGTAAGGAGAGC | 88 | 101.0% | 0.9716 |
|  |  |  |  | TGAAGTCTCAAGTGGGCATG |  |  |  |
|  |  |  | c | CACAGAGACAGAACATTGAG | 63 | 99.2% | 0.9719 |
|  |  |  |  | CTCAAACAGCCAGACCAAAC |  |  |  |
|  |  |  |  |  |  |  |  |
| p15AS | 13 transcripts | 856-3,857 | a | TGCTCTATCCGCCAATCAGG | 58 | 119.9% | 0.9834 |
|  |  |  |  | AGCGGAGCGGCTTTTAGTTC |  |  |  |
|  |  |  | b | TCCCCTCGTCGAAAGTCTTC | 74 | 107.6% | 0.9739 |
|  |  |  |  | ACCTCGCTTTCCTTTCTTCC |  |  |  |
|  |  |  | c | AGAAGAAAACCGGGGAGATC | 65 | 118.6% | 0.9213 |
|  |  |  |  | CTGTTACCTCTGATGGTTTC |  |  |  |
|  |  |  |  |  |  |  |  |
| PANDAR | NR_109836.1 | 1,506 | a | CCTTCGGGTTAAATGTGTGC | 75 | 103.8% | 0.9095 |
|  |  |  |  | TTGGTAACACTGATGCTGAG |  |  |  |
|  |  |  | b | AACTCGGTTTACTACTAGCG | 66 | 157.4% | 0.9190 |
|  |  |  |  | TGATGCCAACCAGATTTGCC |  |  |  |
|  |  |  | c | TTGAATCTGCCTGCATCCTG | 59 | 130.1% | 0.9594 |
|  |  |  |  | GGCAGGAAGGCAAAGAAAAC |  |  |  |
|  |  |  |  |  |  |  |  |
| PVT1 | NR_003367.2 | 1,716 | a | ATGCACTGGAATGACACACG | 65 | 99.0% | 0.9889 |
|  |  |  |  | ACAGCCTCCCTTAAAACCAC |  |  |  |
|  |  |  | b | CTGTTACACCTGGGATTTAG | 62 | 114.1% | 0.9849 |
|  |  |  |  | CAGAGTGCTGAAAGGATATG |  |  |  |
|  |  |  | c | CAGAAGCAATTCAGCCCAAC | 63 | 109.2% | 0.9926 |
|  |  |  |  | TTGGGGCAGAGATGAAATCG |  |  |  |
|  |  |  |  |  |  |  |  |
| UCA1 | NR_015379.3 | 2,314 | a | TGAAGAGATCCACCTGCGAC | 50 | 143.0% | 0.9913 |
|  |  |  |  | ATGTTCCTTGGGCTGGTCTG |  |  |  |
|  |  |  | b | TGGTCACACTTGAAGCAGTC | 60 | 109.8% | 0.9347 |
|  |  |  |  | TCCTTCTGGGGATTACTGGG |  |  |  |
|  |  |  | c | AAACGCTTAGGCTGGCAACC | 54 | 95.7% | 0.9815 |
|  |  |  |  | GTAGGCTTGAGGACACCATG |  |  |  |
|  |  |  |  |  |  |  |  |
| XIST | NR_001564.2 | 19,296 | a | ATTTCTTACTCTCTCGGGGC | 64 | 108.4% | 0.9702 |
|  |  |  |  | AAAGAACCCCAAGTGCAGAG |  |  |  |
|  |  |  | b | AGATTGTGGAGGAAAAGTGG | 60 | 102.2% | 0.9756 |
|  |  |  |  | TAGCCCTAAGCCGAGTTATG |  |  |  |
|  |  |  | c | GTTGTCCACAACCCTATCAC | 59 | 113.1% | 0.9898 |
|  |  |  |  | ACCCCACAGTAATGCAAAAG |  |  |  |
|  |  |  |  |  |  |  |  |
| Yiya | NR_046189.1 | 1,906 | a | TTTTCCTTGTTGCTCTCTCC | 60 | 118.3% | 0.9310 |
|  |  |  |  | AAAGATGTGAGTCCACCCTC |  |  |  |
|  |  |  | b | CCAATCTTTTCCTCCCATAG | 52 | 118.0% | 0.9848 |
|  |  |  |  | ACATTTGGCATTGGGCCTAG |  |  |  |
|  |  |  | c | AGCAACTGAGTGTGAAGATG | 56 | 112.9% | 0.9732 |
|  |  |  |  | TTGCTTCATCTCTGGATGGC |  |  |  |
